# Genome-wide identification and expression analysis of soybean bHLH transcription factor and its molecular mechanism on grain protein synthesis

**DOI:** 10.3389/fpls.2025.1481565

**Published:** 2025-02-19

**Authors:** Nan Wu, Tao Jiang, Yongqi Feng, Minghao Yuan

**Affiliations:** ^1^ School of Agriculture, Jilin Agricultural Science and Technology University, Jilin, China; ^2^ Dongliao County Agricultural Technology Extension Station, Liao Yuan, China; ^3^ Jilin Provincial Seed Management General Station, Changchun, China

**Keywords:** soybean, drought and salt stress, bHLH transcription factor, protein content, molecular mechanism

## Abstract

**Introduction:**

Soybean seeds have a protein content of about 40% and are widely used due to their unique nutritional value. Research has found that drought and nitrogen fertilizer environments are conducive to the formation and accumulation of grain protein. Nitrogen is an essential element for soybean growth and development, and is converted into grain protein through a series of pathways such as the soybean root nodule system. The earliest report on the regulation of nodulation by bHLH transcription factors in leguminous plants was in 2011, but the network regulatory mechanism of their involvement in soybean protein synthesis is still unclear.

**Methods:**

This study we use BLASTP and HMMER to identified 296 soybean GmbHLH genes through whole-genome identification and systematic analysis and is classified into 29 subfamilies, by comprehensively analyzing complex biological issues such as gene structure, function, interactions, and regulatory networks.

**Results:**

This study explores the life processes of soybean growth, development, metabolism, and resistance to adversity.The non-synonymous substitution rate/synonymous substitution rate (Ka/Ks) analysis indicates that most of the homologous genes have undergone purifying selection (Ka/Ks << 1). Cis-acting element analysis of the promoter revealed that this gene family plays an important role in stress response, growth and development, hormone regulation, and other processes. RNA-seq data and qRT-PCR experiments indicated that *GmbHLH* genes were diversely expressed in different organs/tissues, and many *GmbHLH* genes were found to be differentially expressed under salt, and drought stresses, suggesting their critical role in soybean resistance to abiotic stress.

**Discussion:**

The *GmbHLH98* gene(LOC100778376), which is highly expressed under both drought and salt stress, was selected for functional validation. Molecular and agronomic trait analyses of positive transgenic offspring showed that the protein content of soybean seeds increased by 36.8%, indicating that drought and salt conditions promote protein synthesis. This study provides a theoretical basis for exploring the synergistic regulation of drought and salt response and protein synthesis by *GmbHLH98* in the future.These results provide fundamental information about the soybean bHLH genes and will aid in their further functional elucidation and exploitation.

## Introduction

The basic helix-loop-helix (bHLH) transcription factor is a large gene family in plants, with many members playing important roles in plant growth, development, and metabolism. A total of 602 genes have been identified in rapeseed (Ke et al., 2020; Zhao et al., 2020; Li et al., 2020; Wang H. et al., 2020; Yun et al., 2022), while the number of genes in Arabidopsis, mulberry, peanut, and other plants ranges from 100 to 300 (Wu et al., 2022; Dong et al., 2021). The structural domain of bHLH transcription factors contains approximately 60 amino acids, responsible for binding to specific cis-acting elements in the promoter’s basic region at the N-terminus (Yang et al., 2021; Wang et al., 2022b; Huang et al., 2021; Paik et al., 2019; Zhang et al., 2020).The rice bHLH transcription factor *BEAR1* regulates the expression levels of drought-responsive genes such as *LEA3*, *HAK6*, and *NHX4*, which are involved in the rice drought response (Teng et al., 2022). Genomic analysis of foxtail millet indicates that many *bHLH* genes contribute to drought resistance (Qin et al., 2020a). Transcriptomic methods have screened a large number of stress-responsive genes and metabolic pathways related to ion transport and redox function (Peng et al., 2021; Sánchez-Pérez et al., 2019; Shi et al., 2019; Qin et al., 2020b). However, reports on the bHLH transcription factor family genes in soybean under drought and salt stress are relatively scarce among leguminous plants.

The protein metabolism of soybean seeds begins with the process of nitrogen fixation and conversion. Soybeans form a symbiotic nitrogen fixation system with nitrogen-fixing microorganisms, specifically rhizobia, to reduce atmospheric nitrogen into ammonia (Chiluwal et al., 2021; Lyu et al., 2020; Gautrat et al., 2021; Ji et al., 2022). The soybean symbiotic nitrogen fixation system primarily relies on rhizobia (Cai et al., 2020; Chen et al., 2022; Gao et al., 2021). Within the root nodules, rhizobia fix molecular nitrogen from the air and convert it into ammonia. Once molecular nitrogen is reduced to ammonia, it is rapidly transferred to the cytoplasm of root nodule cells (Gautrat et al., 2020; Roy et al., 2020). The fixed ammonia is first synthesized into purines, which are subsequently degraded into ureides. These ureides are then transported through the stem of the soybean to the pod husk, where they are degraded into glyoxylic acid and amino acids. Under the action of key enzymes, these compounds are converted into glutamine and glutamic acid (He et al., 2021; Mu and Chen, 2021; Okuma et al., 2020). After activation, they are transported to mRNA via tRNA for protein polypeptide chain synthesis, elongation, termination, and post-translational modifications, ultimately completing the synthetic metabolism process of soybean protein.

The infection by rhizobia and the formation of root nodules constitute a complex process occurring simultaneously in root hairs and root cortical cells, which is finely regulated by numerous genes. When rhizobia infect legume plants, they induce the activation of many nodulation-related genes, triggering the initiation of infection and the formation of root nodule primordial (Wang et al., 2021). However, the expression of these genes is subsequently rapidly downregulated to prevent excessive nodulation (Sanagi et al., 2021; Zhang et al., 2021; Wang et al., 2022a). Therefore, an in-depth study of the biological functions of bHLH transcription factors that are altered in response to rhizobia and nodulation factors will help us uncover more molecular mechanisms underlying the transcriptional regulation of symbiotic nitrogen fixation in legume plants.

Research has found that the *MtbHLH1* transcription factor in Medicago truncatula can regulate the development of root nodule vascular bundles, thereby exerting a profound impact on nutrient exchange between rhizobia and host plants (Godiard et al., 2011). The bHLH transcription factor *GmPTF1* is involved in regulating nodulation in soybean. This transcription factor binds to the E-box to regulate the expression of the pectin lyase gene *GmNPLa*, which in turn regulates the formation of rhizobial infection threads and enhances the nodulation ability of soybean (Zhang et al., 2024). It is noteworthy that both of these bHLH transcription factors play crucial roles in mature nodules. However, research on the function of bHLH transcription factors during the early stages of nodulation remains scarce. A transcriptome study of Medicago truncatula root hairs in 2023 revealed that the expression of some bHLH transcription factor genes changed significantly within just three days after treatment with rhizobia and their nodulation factors (Ovchinnikova et al., 2023). This finding suggests that bHLH transcription factors can respond to signals from rhizobia and nodulation factors, and actively participate in the infection of rhizobia and the formation of nodules. Additionally, iron homeostasis in soybean is closely related to its biological nitrogen fixation function, as iron is an important component of leghemoglobin. Studies have shown that the bHLH transcription factors *GmbHLH57* and *GmbHLH300* in soybean are both involved in regulating iron homeostasis within the plant. These two transcription factors are expressed in both roots and nodules, and their expression is induced by iron deficiency. When both are overexpressed simultaneously, they trigger the upregulation of downstream iron absorption-related genes, leading to an increase in iron content within the plant and enhancing its tolerance to iron deficiency stress (Wu et al., 2023; Wang W. et al., 2020).

The expression level of bHLH transcription factors can affect the number and quality of nodules in plants. By regulating the expression of related genes, bHLH transcription factors can promote or inhibit the formation of nodules, thereby influencing the absorption and utilization of nutrients such as nitrogen by plants. Apart from bHLH transcription factors, there are multiple other transcription factors involved in the nodulation process of plants. NAC transcription factors perform various functions in plants, including regulating plant growth, development, and responses to environmental stresses. During nodulation, NAC transcription factors promote the formation of root nodules and symbiotic nitrogen fixation by activating the expression of relevant genes. In soybeans, the NAC transcription factor *GmNAC181* activates *GmNINa* and the nodulation signaling pathway to facilitate nodulation. Furthermore, *GmNAC181* acts as a molecular network node, coordinating the plastic development of symbiotic nodulation under salt stress (Wang et al., 2022a). This discovery not only provides data support for elucidating the molecular mechanisms underlying soybean’s salt-tolerant and stable nitrogen fixation but also offers new insights into the cultivation of soybean varieties with high nitrogen fixation efficiency, stable nitrogen fixation under salt stress, and consistent high yields.

Drought and salt stress have significant effects on soybean protein synthesis, and these effects vary depending on the stress conditions, growth stages, and variety characteristics. To optimize the quality and yield of soybeans, it is necessary to delve into the physiological and molecular mechanisms underlying these stress conditions. Meanwhile, a diverse array of transcription factors are involved in plant nodulation, which jointly affect the infection of rhizobia, the formation of root nodules, and the efficiency of symbiotic nitrogen fixation through complex regulatory networks. In this study, 296 non-redundant soybean (Glycine max) bHLH genes were identified using bioinformatics analysis on a genome wide scale and molecular biology techniques. We used online MEME software, TBtools, PlantCARE and Phytozome v13 website to obtain gene structure, conserved motifs, chromosome localization, phylogenetic analysis, cis acting elements, and expression profiles under different tissues and abiotic stresses. Currently, genome-wide expression analysis has taken center stage as a vital strategy for uncovering and examining gene functions. This approach involves identifying and elucidating the intricate biological processes that are either integral to or governed by specific target genes.G*mbHLH98*, which was significantly upregulated under drought and salt stress, conditions was chosen for further analysis. Overexpression of *GmbHLH98* improved soybean tolerance to drought and salt stress, indicating the importance of the *GmbHLH98* gene in abiotic stress responses. Meanwhile, under drought and salt stress, the protein content of genetically modified seeds increased. In the future, through methods such as CRISPR, overexpression, and protein interaction, we will provide a theoretical basis for further exploring the synergistic regulatory network mechanisms of bHLH transcription factors in abiotic stress and plant nodulation symbiotic nitrogen fixation.

## Materials and methods

### Plant materials, growth environment, and stress treatment

T2 generation transgenic soybean plants (OE1, OE2, KO1, KO2) were pre-selected and constructed using the overexpression vector pCAMBIA3301-GmbHLH98 and the gene editing vector pCRISPR-GmbHLH98 from the control variety Jike Soybean 20 (WT) at Jilin Agricultural Science and Technology University. The seedling stage testing method follows Zhou et al. (2023) and Zhang et al. (2022). Selected seeds were plump and insect-free, disinfected with 75% alcohol and 5% NaClO, and germinated in pots. The artificial climate chamber conditions were set to 25°C (day)/16°C (night), with a 16/8 hour light/dark cycle and 75% relative humidity. Drought stress was applied using 200 mmol/L PEG6000, and salt stress with 200 mmol/L NaCl, while the control group received normal field management and distilled water. Soybean leaves were collected at 0, 4, 8, 12, 24, and 36 hours, frozen in liquid nitrogen, and RNA was extracted from young leaf tissues. Three biological replicates were used for each time point. Soybean seedlings at the V2 stage were subjected to stress conditions (200 mmol/L PEG6000 and 200 mmol/L NaCl), and root system growth was documented through photography.

### Identification of *GmbHLHs* gene family in soybean

Genome data and gff annotation information for the soybean genome (GCF-00004515.6-Glycine_max_v4.0) were downloaded from the NCBI website (https://www.ncbi.nlm.nih.gov/). The amino acid sequence of the Arabidopsis bHLH gene was obtained from the Arabidopsis thaliana information website (TAIR) (https://www.arabidopsis.org/). The Blast Compare Two Seqs module in TBtools v2.101 was used to set the E-value to 10^-5 for local BLAST. Additionally, the Pfam gene database (http://pfam.xfam.org/) was searched for the characteristic structural domain (PF00010) of the soybean *bHLH* gene family. Application of Hidden Markov Model Search Tool (HMMER 3.3.2 version; http://hmmer.org/) and Basic Local Alignment Search Tools for Proteins (BLASTP; https://blast.ncbi.nlm.nih.gov/Blast.cgi) used for searching soybean genome(Wm82.a4.v1). The intersection of the sequence IDs was filtered twice, and the online bioinformatics tool National Center for Biotechnology Information CDD (https://www.ncbi.nlm.nih.gov/Structure/bwrpsb/bwrpsb.cgi) and the SMART Online Website (https://smart.embl.de/smart/set_mode.cgi?NORMAL=1) were used to verify the presence of a conserved bHLH domain. Finally, using CD-HIT software, we set 90% homology to remove redundant sequences and manually retained only the longest transcript sequences for different transcripts of the same gene.

### Phylogenetic analysis

The amino acid sequences of *bHLH* genes from soybean and Arabidopsis thaliana were selected for phylogenetic analysis, using Linum usitatissimum with the default parameters. Clustal was used for multiple sequence alignment, and after alignment, MEGA11 software was used with the Neighbor Joining (NJ) method to construct a phylogenetic tree, setting the Bootstrap value to 1000. Afterwards, the evolutionary tree was visualized and enhanced using iTOL (https://itol.embl.de/).

### Analysis of gene structure and conserved motif, chromosome localization, cis-acting elements of *GmbHLHs* genes

The motif structure of soybean bHLH family members was analyzed using online MEME software (http://www.omicclass.com/article/67), setting the maximum number of motifs to 20, the minimum width to 6, and the maximum width to 50. The structure of the soybean *bHLH* gene, including CDS and UTR, was extracted from the soybean genome annotation file. For visual analysis, TBtools v2.101 was used to extract chromosome position information of soybean *bHLH* genes from the genome annotation files, and it was visualized using the Gene Location Visualize feature in the GTF/GFF module of TBtools. The 2000 bp region upstream of the start codon (ATG) of each soybean *bHLH* gene is considered a promoter sequence. The promoter sequence was extracted using TBtools and predict cis-regulatory elements using PlantCARE (https://bioinformatics.psb.ugent.be/webtools/plantcare/html/analysis). Cis-elements related to stress response, plant growth and development, plant hormone response, and light response were visualized and summarized using the HeatMap module in TBtools.

### Covariance analysis and gene replication of the soybean *bHLH* gene

The BLASTP program is employed to identify homologous *bHLH* genes in soybean, with an e-value threshold set to < e^−5^. Use MCScanX with default parameters to analyze the collinearity relationships between soybean *bHLH* genes, visualizing the results with TBtools.

For collinearity analysis, refer to the Phytozome v13 website (https://phytozome-next.jgi.doe.gov/) to download Arabidopsis and rice genome sequences and annotation files from the NCBI website (https://www.ncbi.nlm.nih.gov/), as well as the corn genome sequence and annotation files from the Sol Genomics Network (https://solgenomics.net/organism/solanum_lycopersicum/genome). The collinearity between soybean and *Arabidopsis*, corn, rice, and tobacco was analyzed using the MCScanX program.

Calculate the nucleotide synonymous (Ks) and non-synonymous (Ka) substitution frequencies and Ka/Ks values using the TBtools software Set Ka/Ks_Calculator and perform Ka/Ks analysis. According to Darwin’s theory of evolution, Ka/Ks>1.0 indicates positive selection,Ka/Ks < 1.0 indicates that purification selection occurs, and Ka/Ks = 1.0 indicates neutral selection. In addition, the divergence time (T) is calculated using the formula. T= Ks/(2×6.1×10^−9^) calculated in millions of years ago (Mya)(Fan et al., 2013).

### Three-dimensional protein structure prediction and GO enrichment analysis

The protein secondary structure of GmbHLH was predicted using GOR IV (https://npsa-prabi.ibcp.fr/cgi-bin/npsa_automat.pl?page=/NPSA/npsa_gor4.html). The online SWISS-MODEL tool was utilized to predict the 3D structure of the proteins. The go-base.obo file was downloaded in TBtools, and the protein information for the entire soybean genome was inputted into the online EggNOG Mapper (http://eggnog-mapper.embl.de/) to obtain the annotation file. The GO Enrichment feature in TBtools was used for visualization.

### Expression patterns of *GmbHLHs* genes in different tissue parts of soybean

Soybean RNA-seq data were obtained from the Phytozome database (https://phytozome-next.jgi.doe.gov/). This dataset includes nine tissues and organs: nodal root, seed at 10 DAF, seed at 14 DAF, pod shell at 10 DAF, pod shell at 14 DAF, one cm pod, flower, and young leaf. The expression level of each gene is represented as fragments per kilobase of transcript per million mapped reads (FPKM), and the transformed values (FPKM + 1) of the *GmbHLHs* genes were generated using log2 software.

### RNA extraction and qRT-PCR analysis

RNA was extracted from tender soybean leaves subjected to drought and salt stress treatments using an RNA extraction kit (TSINGKE, cat. TSP401, China). After freezing in liquid nitrogen, the leaves were quickly ground into a powder. A total of 200 mg of the powder was weighed and mixed with 0.5 μL of buffer RL. The RNA was purified using RNase-free columns and finally eluted in 30 μL of RNase-free ddH_2_O. Following concentration detection, the RNA was reverse transcribed into cDNA and stored at −40°C for future use. SYBR Green™ Premix ExTaq™ II (TaKaRa Bio, Kyoto, Japan) was used for fluorescence quantification in qRT-PCR with a LightCycler 480 instrument (Roche). The PCR reaction system comprised 20 μL, which included 10 μL of 2× mix, 1 μL each of upstream and downstream primers (**Supplementary Table S4**), 2 μL of cDNA template, and 6 μL of sterile water. The qRT-PCR program was conducted as follows: 50°C for 2 minutes, 95°C for 10 minutes, followed by 45 cycles of 95°C for 15 seconds, 60°C for 15 seconds, and 72°C for 15 seconds. *β-Actin* (GenBank accession number: NM_001252731.2) was selected as the internal reference gene. The relative gene expression was calculated using the 2^−ΔΔCt^ method, and SPSS 19.0 software (SPSS Inc., Chicago, IL, USA) was utilized for data analysis. To assess the reproducibility and stability of our experiment, three biological replicates were selected, and the data are presented as mean ± standard error.

### *GmbHLH98* subcellular localization analysis

RNA was extracted from young soybean leaves, reverse transcribed into cDNA, and used as a PCR template to clone the full-length *GmbHLH98* gene. Primer 5.0 software was used to design primers (**Supplementary Table S4**). GFP and *GmbHLH98* were ligated into the pCAMBIA1302 vector to obtain pCAMBIA1302-GFP and pCAMBIA1302-GmbHLH98-GFP, in plant subcellular localization experiments, Agrobacterium tumefaciens is often used as a vector to introduce target genes into plant cells. All experiments were independently replicated in triplicate. The bacterial suspension was incubated in infection buffer at 28°C for 3 hours, injected into tobacco leaves using a syringe, and incubated in the dark for 3 days. The position was observed using a confocal microscope.

### Construction of expression vectors and genetic transformation

CRISPR-P (http://cbi.hzau.edu.cn/CRISPR2/help.php) was utilized to design a target for an online website and construct a single gene dual-target vector for the target gene. The target sequence primarily consists of 19 bases alongside a PAM structure, which facilitates Cas9 binding with gRNA. The reaction system comprised the following components: 2 µL of CRISPR/Cas9 vector, 2 µL of oligomers, 1 µL of enzyme mixture, topped up with deionized water to a total volume of 10 µL. All reactions were conducted on ice and then mixed and allowed to react at room temperature. The vials were incubated at 20°C for 1 hour, followed by subjecting to transformation into *E. coli* competent cells, extraction of plasmid DNA, and Sanger sequencing. Primer 5.0 was used to design overexpression primers for the target gene. These are then linked to the pCAMBIA3301 vector and subjected to double-enzyme digestion to verify whether the construction was successful. The resultant vectors are named pCAMBIA3301-GmbHLH98 and pCRISPR-GmbHLH98, respectively.

Using the soybean variety Jike Soybean 20 as the receptor material, the above vector was transferred into the receptor soybean variety through an *Agrobacterium tumefaciens*-mediated method. The steps include pre-cultivation, co-cultivation, screening, differentiation, rooting, seedling refinement, and transplantation to obtain T2 generation transgenic soybean plants. PCR detection was performed using the specific primers Cas9-98F/Cas9-98R and Bar-1F/Bar-1R (**Supplementary Table S4**). The PCR conditions are as follows: a total volume of 20 µL, starting with an initial denaturation at 94°C for 5 minutes; followed by 30 cycles of 94°C for 30 seconds, 60°C for 30 seconds, and 72°C for 10 minutes.

### Evaluation of transformation efficiency and gene-editing efficiency

Primers were designed based on templates encompassing the 200 bp regions upstream and downstream of the gRNA, specifically: Target 1: gRNA-98F1/gRNA-98R1; Target 2: gRNA-98F2/gRNA-98R2 (**Supplementary Table S4**). PCR reactions were conducted using GoTaq^®^ G2 Green Master Mix (Promega). The PCR products were electrophoresed on a 1% agarose gel and visualized using the Gel Doc™ EZ imaging system. Leaf DNA was extracted as the template for assessing gene editing efficiency. The PCR amplification products of the bHLH98-sgRNA1 target site were digested with *Bst*EII, while those of the bHLH98-sgRNA2 target site were digested with *Hin*dIII. After amplification using target-specific primers, the products were recovered and sent to Sangon Biotech (Shanghai) Co., Ltd. for Sanger sequencing. Snapgene software was utilized to analyze the gene editing status of each gRNA target site, and the gene editing efficiency and mutation types for each vector were recorded. The amino acid sequences of the mutants were aligned using the online tool Job Dispatcher (https://www.ebi.ac.uk/jdispatcher/), with base changes in the gRNA sequences indicating successful target gene editing. Furthermore, T_2_ generation plants were confirmed to be Cas9-free, and mutation types were determined based on the alignment results.

### Seedling experiment under drought and salt stress

The seedling stage test method follows the protocols outlined by Zhou et al. (2023) and Zhang et al. (2022). Seeds of the control variety Jike Soybean 20, overexpression material, and gene editing material are disinfected with 75% alcohol and 5% NaCIO, then placed in flower pots for germination. Drought stress is induced with 200 mmol/L PEG6000, and salt stress with 200 mmol/L NaCl. The control group receives normal field management, including the same amount of distilled water. All materials germinate in an artificial climate incubator set to a constant temperature of 25 °C and a relative humidity of 70%. Soybeans germinate about 7 days (VE stage) after sowing, with root growth observed and photographed on the 25th day (V2 stage) after emergence.

### Determination of protein content in T_2_ generation soybean seeds by near-infrared grain analyzer

Near-infrared grain analysis is the most commonly used spectral analysis technique, capable of establishing a measurement model based on a database and performing subsequent quality content determination. In this experiment, the NIRS DS2500 grain analyzer was utilized. The selection of grains was based on cleanliness; mature, complete, and insect-free seeds were placed into a measuring cup, covering the bottom of the cup and the infrared scanning area. Each sample was measured three times to minimize errors. The measurement results were collected and analyzed through software, with the results automatically saved to the computer. A survey was conducted on the agronomic traits of the positive material and the control variety.

### Analysis of main agronomic traits

This study employed a field planting method, sowing at the end of April using a hole sowing technique. Each ridge was 3 meters long with an interval of 15 cm, sowing 2 seeds per hole, with soybean growth occurring from April to October.

The determination of soybean yield primarily includes the number of pods per plant and the weight of 100 seeds per plant. The weight of soybean plants is determined by measuring the weight (g) of 100 seeds per plant. The specific method involves randomly selecting 5 plants from the middle row under the same treatment conditions and weighing 100 randomly selected seeds from the total number of beans; this process is repeated three times. Other agronomic traits assessed include plant height, number of branches, and number of stem nodes. We measured the plant height (cm) of positive soybean plants by randomly selecting 5 plants from the middle row and using the unearthed position as a reference. Measurements were taken from the top of the plants with a ruler, repeating the measurement three times. These data were analyzed using IBM SPSS statistical software.

### Statistical analysis

All experiments in this study were repeated three times. We used SPSS version 19.0 (SPSS Inc., Chicago, IL, USA) to analyze the significance of differences in protein content of T2 soybean seeds using Duncan’s multiple range test.

## Results

### Identification of *GmbHLH* genes in soybean

**Figure 1:**
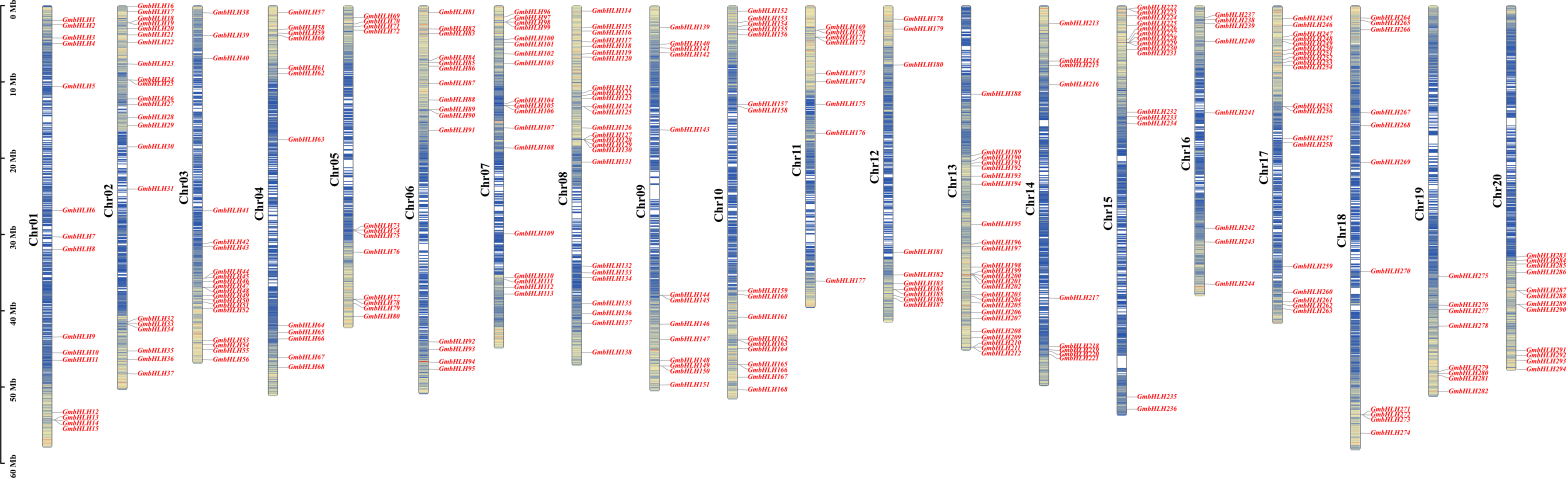
The location of GmbHLHs family members on chromosome. Chromosome numbers are on the left and GmbHLHs are on the right of chromosomes. Scale bar on the left indicates chromosome length.

According to the NCBI website (https://www.ncbi.nlm.nih.gov/), downloading the genome data and gff annotation information for the soybean genome (GCF-00004515.6-Glycine_max_v4.0) yielded a total of 296 soybean *GmbHLH* genes. Based on the gff3 file of soybean genome annotation, these 296 *GmbHLH* genes were located on 20 soybean chromosomes and named *GmbHLH1* to *GmbHLH296* (**Figure 1**) according to their chromosomal positions. Chromosomes 8 and 13 had the highest distribution, each containing 25 genes, which accounts for 8.4% of the total. Chromosome 2 follows with 22 genes, representing 7.4% of the total, while chromosome 17 contains 19 genes, accounting for 6.4%. In contrast, chromosome 19 has the fewest genes, with only 8, or 2.7% of the total.

The physicochemical propeies of soybean bHLH proteins indicate that their lengths range from 92 amino acids (*GmbHLH64*) to 965 amino acids (*GmbHLH206*), with theoretical isoelectric points varying between 4.516 (rna-NM001248975.2) and 11.819 (rna-XM003550868.4). Among the 215 bHLH proteins analyzed, most have isoelectric points between 4 and 7, suggesting that the majority of soybean bHLH family proteins are theoretically acidic (**Supplementary Table S1**).

### Gene structure and conserved motif analysis of the soybean *bHLHs* family

**Figure 2:**
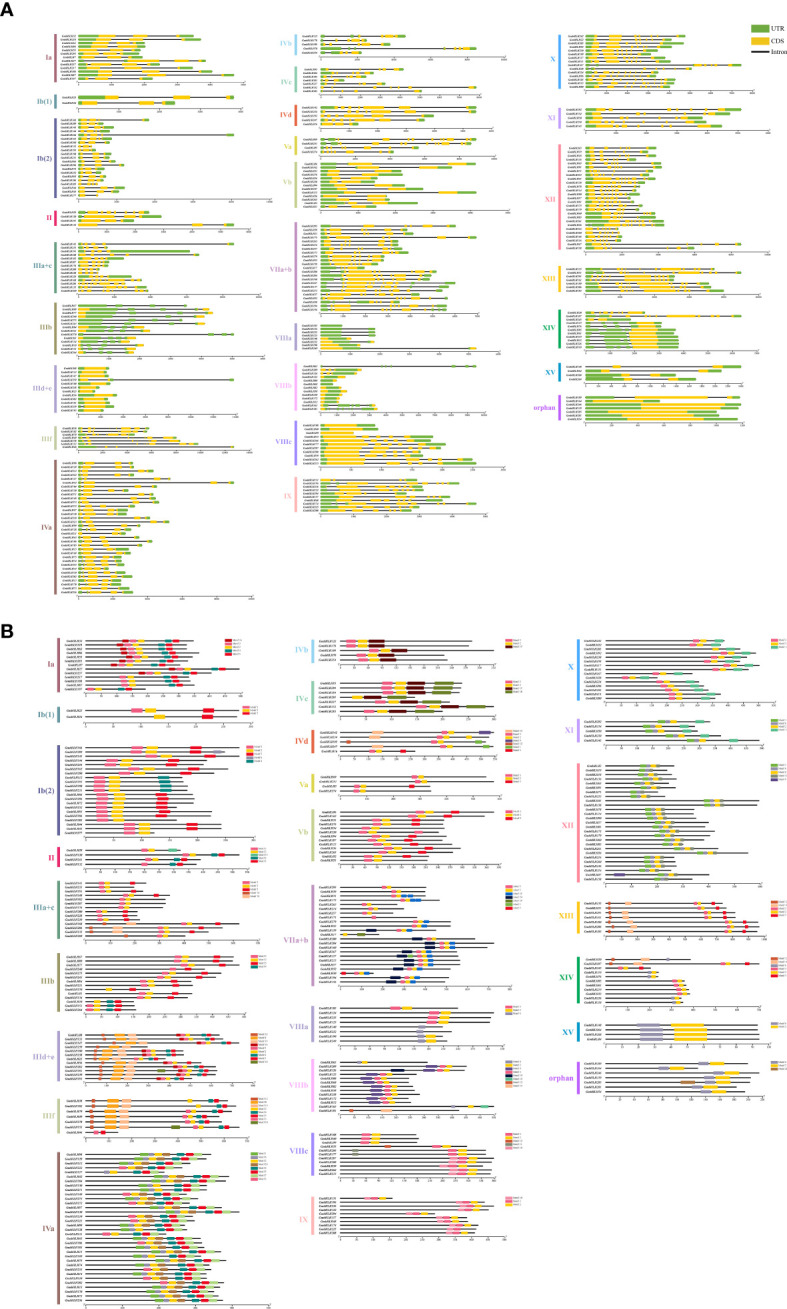
Gene structure of GmbHLHs **(A)** and conserved motifs of GmbHLHs **(B)** in soybean. **(A)** Structural analysis of the CDS and intron boundaries of the GmbHLH family: The yellow box indicates the CDS region, the black line indicates the intron, and the green box indicates the UTR. **(B)** Putative motifs in each GmbHLH protein. Conserved motifs were identified using MEME and TBtools software. Twenty putative motifs are indicated by colored boxes. The length of each protein can be estimated using the scale at the bottom.

Gene structure and conserved motif analysis can help explore the evolutionary relationships and functional basis of soybean bHLH family members. All 296 *GmbHLH* genes contain multiple exons and introns, with the number and structural composition of introns and exons being roughly similar among genes located within the same branch of the evolutionary tree. The number of introns varies from 0 to 10; 28 genes have no introns, accounting for 9.5% of the total, while 18 genes contain one intron, representing 6.1% of the total. The remaining genes have two or more introns, with the number of exons ranging from 0 to 11. Seven genes have no exons (2.4% of the total), and one gene (0.3%) contains 11 exons (**Figure 2A**). To determine the composition and quantity of conserved motifs in the *GmbHLH* gene family, we utilized MEME online software, which identified a total of 20 conserved motifs labeled Motif1 to Motif20. Each *GmbHLH* protein contains a varying number of conserved motifs, ranging from 2 to 8, with most bHLH members sharing common motifs. All 296 (100%) *GmbHLH* proteins contain Motif 2. Additionally, the conserved motifs in the IIId+e group (*GmbHLH8/133/147/259/100/238/23/56/282/101/239/193*) are the most complex, containing 9 conserved motifs. The diversity of the soybean *bHLH* gene family relates to the distribution and structure of conserved motifs, indicating that genes within the same branch possess similar sequences and motifs (**Figure 2B**).

### Phylogenetic analysis of the soybean bHLHs family

**Figure 3:**
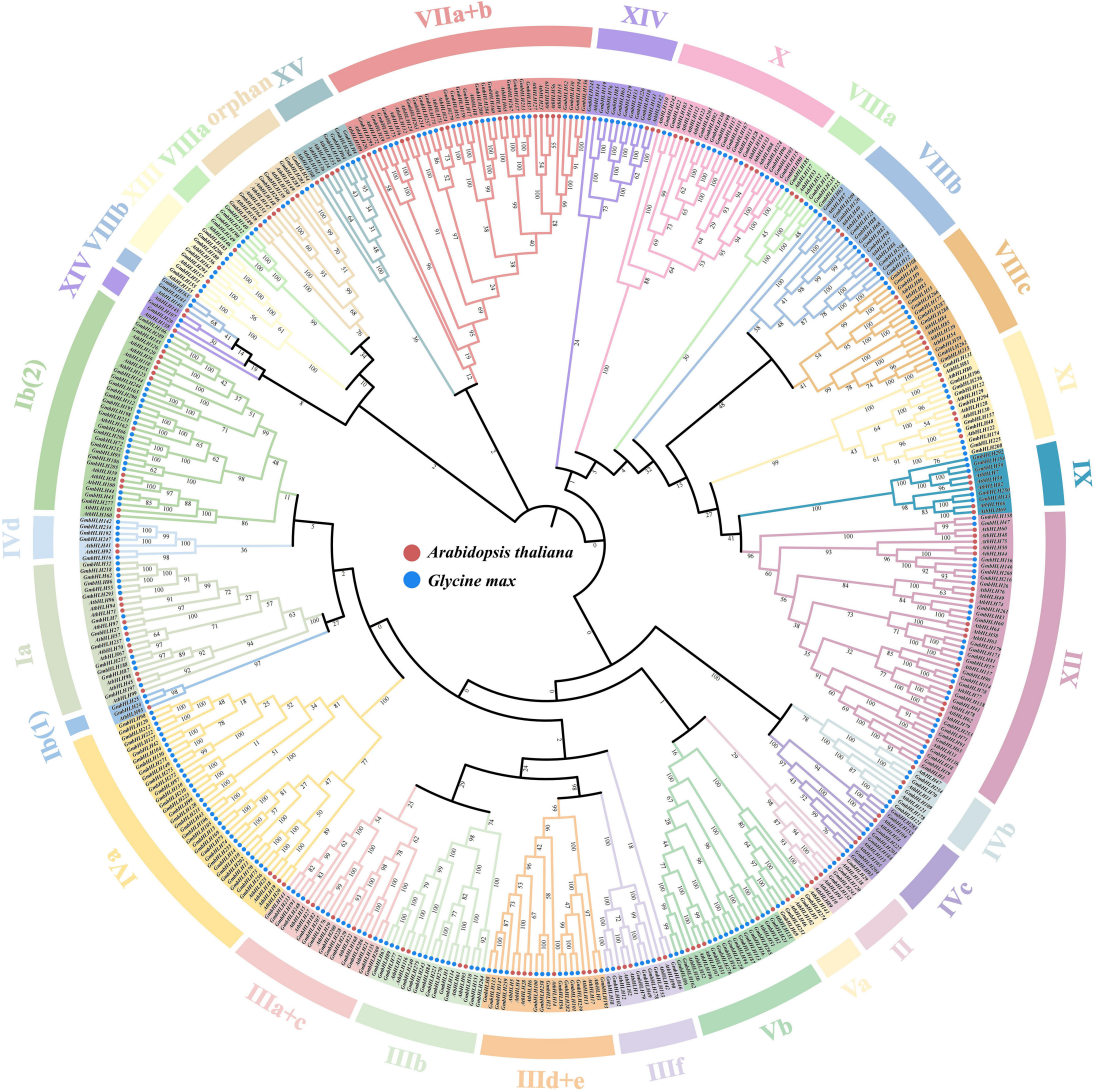
Phylogenetic tree of the soybean GmbHLHs protein. Phylogenetic analysis of bHLH proteins from Glycine max and Arabidopsis thaliana. 159 AtbHLHs and 296 GmbHLHs were aligned with the Clustal W program, and a neighbor-joining phylogenetic tree with 1000 bootstrap replications was constructed using the MEGA 11.0 software.

To study the evolutionary relationship between soybean and Arabidopsis thaliana bHLH genes, this study used MEGA11 and its built-in ClustalW to perform systematic evolutionary analysis on the amino acid sequences of 296 soybean GmbHLHs and 159 AtbHLHs (**Figure 3**). The results showed that the bHLH proteins of the two species were divided into 29 subfamilies, with the XII group containing the largest number of members (44 proteins), while the Ib(1) group and VIIIb group contained the fewest members (only 3 proteins each). Both species contain these subfamilies, indicating that the functions of soybean and Arabidopsis thaliana subfamily members are relatively conserved. All bHLH genes in group X contain 4-5 introns; Three genes have four introns and five genes have five introns. Previous studies have shown that different subfamilies regulate the growth and development of plant roots, stems, axillary buds, etc., participate in gibberellin, light signal, brassinolide and other signal transduction pathways, and respond to plant responses to drought, salt stress and other important effects. It is speculated that the GmbHLHs proteins of different subfamilies in soybeans may play an important role in regulating their own growth and development, stress response, etc.

### Collinearity analysis of soybean *bHLHs* protein family

Gene replication events primarily include tandem duplication and segmental duplication, which can increase the number of gene family members and are significant for promoting new gene formation, diversifying gene functions, and driving evolution. Collinearity analysis of soybean *GmbHLH* family genes showed that genes involved in segmental duplication were connected by red curves (**Supplementary Figure S1**). Analysis of gene positions revealed that some genes on chromosome 13 did not undergo duplication events, indicating that in soybean *GmbHLH* gene amplification, the segmental duplication rate is greater than the tandem duplication rate. The intron-exon structures and conserved motifs of each pair of duplicated genes are similar, highlighting that gene duplication has played a crucial role in the expansion and evolution of the *GmbHLH* family. To assess the selective pressure on soybeans during evolution, homologous genes of the bHLH family in soybean species were used to calculate Ka (non-synonymous mutation rate), Ks (synonymous mutation rate), and the value of Ka/Ks. The results showed that soybean bHLH gene family. The Ka/Ks values of all genes in the *GmbHLH* family are significantly less than 1.0(0.0466 to 0.7106) (**Supplementary Table S2**), suggesting that the soybean *GmbHLH* family genes may have undergone non-conservative mutations. The negative selection of Changqiang to maintain their functions indicates that these genes have high conservation and structural stability during the evolutionary process. Stability and functional consistency. In addition, in order to understand the evolutionary history of the soybean *GmbHLH* gene family, the divergence time (T) was also calculated. The separation of soybean bHLH genes occurred between 5.64-302.90 million years ago.

### Interspecies collinearity analysis of the soybean *GmbHLH* gene family

**Figure 4:**
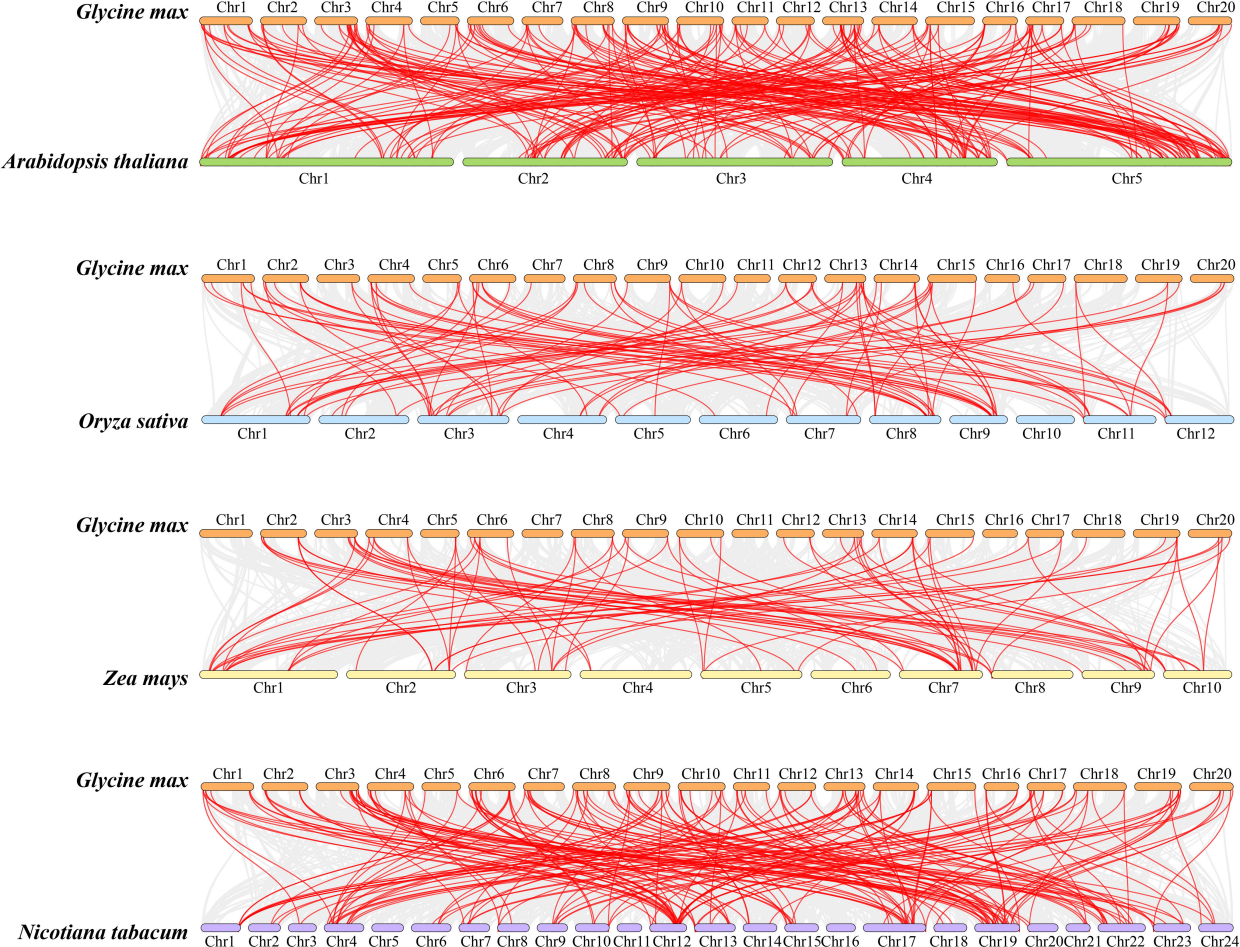
Collinearity analysis of the bHLH gene family between soybean and Arabidopsis, maize, rice, and tobacco. Gray lines represent the collinear blocks between two plants in their genome. Syntenic gene pairs are represented by the marked red lines.

Further analysis was conducted on the collinear relationships between members of the soybean *GmbHLH* gene family and *bHLH* genes in Arabidopsis, rice, tobacco, and maize. A collinearity plot was created to illustrate the homologous gene pairs between soybean and these four plants, connected by red lines (**Figure 4**). The abundance of bHLH homologous gene pairs indicates that soybean and Arabidopsis share the highest homology, followed by tobacco, rice, and maize. The maximum number of homologous genes with Arabidopsis chromosome 5 is 62; with rice chromosome 3, it is 17; with maize chromosome 1, it is 19; and with tobacco chromosome 19, it is 23. The intron-exon structures and conserved motifs of each pair of duplicated genes are similar, reinforcing the importance of gene duplication in the expansion and evolution of the *GmbHLH* family.

### Analysis of cis acting elements in the promoter of soybean *GmbHLH* family genes

The cis-acting elements of promoters play a crucial role in the transcriptional regulation of gene expression, reflecting environmental factors and tissue specificity. The promoter sequence of the *GmbHLHs* gene, located upstream of the start codon, was extracted using TBTOOLS software, and the cis-acting elements were analyzed using the PlantCARE database. The results (**Supplementary Figure S2**) revealed that the promoter sequence of *GmbHLHs* contains numerous elements related to stress responses, hormones, and plant growth and development. However, the types and quantities of these elements vary among different *GmbHLHs* genes. The bHLH transcription factor primarily participates in plant drought stress responses by recognizing E-box elements, with the most commonly bound E-box being the G-box (5’-CACGTG-3’). Notably, 75.7% of the genes in the soybean *GmbHLHs* family contain ABRE (5’-ACCCGG-3’) elements, suggesting that the expression of these *GmbHLHs* genes is regulated by their family members. Genes containing the drought-inducing element MBS account for 35.2%; among these, *GmbHLH17, GmbHLH23, GmbHLH119, GmbHLH120, GmbHLH198, GmbHLH202*, and *GmbHLH267* contain three MBS elements, while *GmbHLH42* and *GmbHLH213* contain four. Additionally, genes with low-temperature responsive elements LTR account for 27.1%, and those with the low-temperature element P-box account for 33.5%. Among the *GmbHLHs* genes containing hormone-responsive elements, 27.1% have auxin TGA elements/TGA box elements, 75.7% contain abscisic acid (ABA) responsive ABRE elements, 42.3% contain salicylic acid responsive SAER/TCA elements, 67.3% have methyl jasmonate (MeJA) responsive CGTCA motif/TGACG motif elements, and 33.5% contain gibberellin responsive P-box/TACbox/GARE motif elements. This indicates that many *GmbHLHs* genes are involved in hormone signaling pathways. Furthermore, 57.5% of the *GmbHLHs* gene promoters contain the damage response element WUN motif, while 20.3% include the grain storage protein GCN4_motif element (**Supplementary Table S3**).

### *GmbHLHs* protein interaction network and GO enrichment analysis

**Figure 5:**
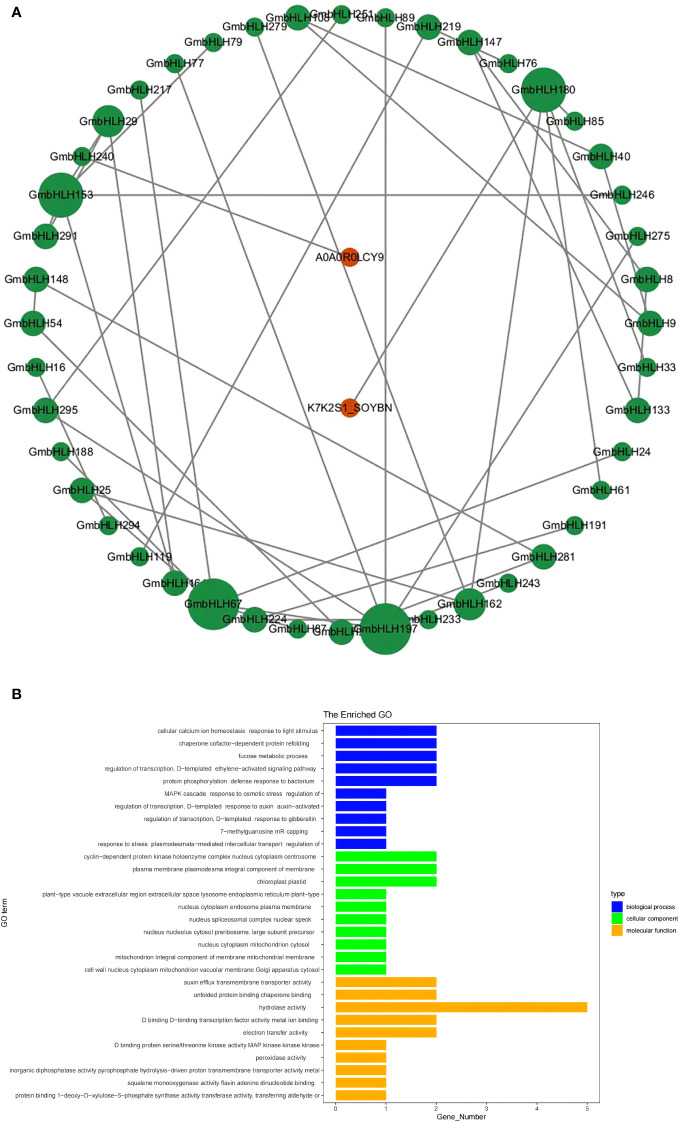
Protein-protein interaction network **(A)** and GO enrichment analysis **(B)** of soybean *GmbHLHs* gene family. **(A)** Constructing a protein interaction network of the *GmbHLHs* gene family through *Arabidopsis* orthologs. The green circle represents soybean GmbHLHs protein, and the red circle represents the reported genes in *Arabidopsis*. **(B)** GO enrichment analysis of *GmbHLHs* gene.

To further investigate the potential biological functions of the *GmbHLHs* gene family, orthologous genes from Arabidopsis were used to predict the protein interaction network of the *GmbHLHs* family (**Figure 5A**). The analysis showed that orthologs of 48 *GmbHLHs* genes were predicted to interact with other proteins, some of which are associated with stress responses or flower development. GO enrichment analysis was performed on the *GmbHLH* gene family across biological processes, cellular composition, and molecular function (**Figure 5B**). The biological processes primarily enriched included transcriptional regulation, cellular calcium homeostasis, dependent protein partner refolding, co-metabolic processes, protein phosphorylation, and defense responses. The cellular composition was mainly enriched in cyclin-dependent protein kinase complexes, intercellular filaments, chloroplasts, and protoplasts. The molecular functions primarily enriched included hydrolytic enzyme activity, auxin transmembrane transport, binding to unfolded proteins, binding to transcription factors, and electron transfer.

### *GmbHLHs* expression pattern analysis based on RNA-seq data

**Figure 6:**
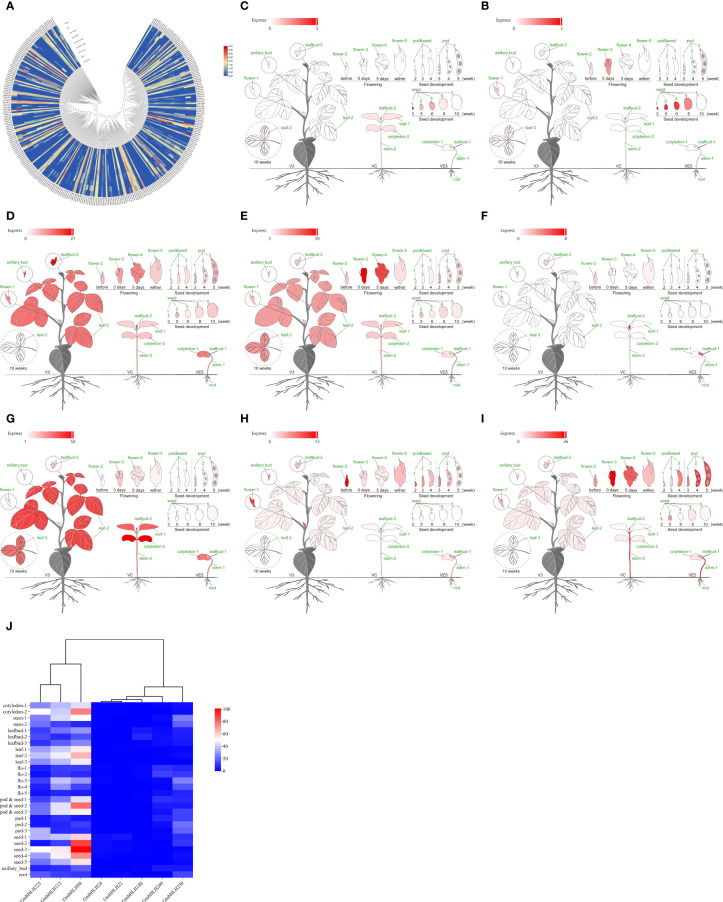
**(A)** Analysis of expression patterns of soybean *GmbHLHs* gene in different tissue parts of soybean. **(B–I)** Display the expression pattern of gene *GmbHLHs* in different parts of soybean: **(B)** *GmbHLH22、* **(C)** *GmbHLH24、* **(D)** *GmbHLH98、* **(E)** *GmbHLH115、* **(F)** *GmbHLH188、* **(G)** *GmbHLH225、* **(H)** *GmbHLH249、* **(I)** *GmbHLH250*. Red represents high expression; gray represents low expression. **(J)** Heatmap was drawn by Chiplot. Red represents high expression, and blue represents low expression.

The *GmbHLHs* gene plays a crucial role in plant growth, development, and response to abiotic stress. We analyzed the expression patterns of the identified soybean *GmbHLHs* gene family using RNA-seq data (**Figure 6A**). The analysis revealed diverse expression patterns across different tissue sites: 73 *GmbHLHs* genes were highly expressed in soybean root nodules. The genes showing high expression in soybean roots include GmbHLH56*, GmbHLH60, GmbHLH98, GmbHLH115, GmbHLH202*, and *GmbHLH245*; in seeds, *GmbHLH249, GmbHLH232, GmbHLH98, GmbHLH188, GmbHLH22, GmbHLH24, GmbHLH173, GmbHLH114, GmbHLH287*, and *GmbHLH250*; in flowers, *GmbHLH61, GmbHLH225, GmbHLH249, GmbHLH232, GmbHLH115, GmbHLH96, GmbHLH22, GmbHLH250, GmbHLH123, GmbHLH98, GmbHLH25*, and *GmbHLH188*; and in leaves, *GmbHLH115, GmbHLH225, GmbHLH22, GmbHLH292, GmbHLH123, GmbHLH98, GmbHLH24*, and *GmbHLH250*. This gene family plays a vital role throughout the various stages of soybean growth and development. To capture the diversity of the *GmbHLHs* family, we selected 8 highly expressed genes (*GmbHLH22, GmbHLH24, GmbHLH98, GmbHLH115, GmbHLH188, GmbHLH225, GmbHLH249, GmbHLH250*) from the transcriptome data for further experimental studies (**Figures 6B-J**).

The Phytozome dataset encompasses roots, root tips, lateral roots, stems, stem tips, leaves, flowers, hypocotyls, compound leaves, pods, seeds, and lateral buds. Many *GmbHLH* genes are preferentially expressed in multiple tissues, with *GmbHLH98* and *GmbHLH115* being notably highly expressed, whereas *GmbHLH22* and *GmbHLH24* are either lowly expressed or undetected in the dataset. The data also indicates that over 40% of highly expressed *GmbHLH* genes are found in soybean seeds.

### Expression levels of *GmbHLHs* genes under abiotic stress

To ensure representation of the diversity of the *GmbHLHs* family, we selected highly expressed genes from earlier transcriptome data for subsequent experiments. These selected genes showed high homology with known genes in Arabidopsis. Additionally, qRT-PCR was utilized to evaluate the relative expression levels of these genes to assess the *GmbHLHs* gene’s response to abiotic stress. We studied the relative expression of 8 genes (*GmbHLH22, GmbHLH24, GmbHLH98, GmbHLH115, GmbHLH188, GmbHLH225, GmbHLH249, GmbHLH250*) in root, stem, and leaf tissues under drought and salt stress (**Supplementary Figures S3**, **S4**), across different subgroups. Gene expression levels were compared at intervals of 0, 4, 8, 12, 24, and 36 hours, showing significant responses with increasing stress duration.

Drought treatment induced significant upregulation of the expression of two genes (*GmbHLH98, GmbHLH115*), while *GmbHLH24* and *GmbHLH250* exhibited a downward trend. The expression levels of other genes did not show significant changes (**Supplementary Figure S3**). The transcription levels of GmbHLH98 in root, stem, and leaf tissues peaked at 8 hours of drought stress and then gradually decreased, showing an increase of 13-17 times compared to the control group. *GmbHLH115* in root and stem tissues followed a similar expression pattern, reaching their maximum at 24 hours of drought stress, with increases of 3.15 times and 3.09 times, respectively, compared to the control group. Leaf tissue exhibited a significant downward trend at 36 hours of drought stress. The transcription levels of *GmbHLH24* in root, stem, and leaf tissues reached their lowest values after 36 hours of drought stress, decreasing by 27.5 times, 85 times, and 36.7 times, respectively. *GmbHLH250*’s transcription levels in root and stem tissues also reached their lowest values at 36 hours of drought stress, decreasing by 6.1 and 5.8 times, respectively. The transcription level of leaf tissue reached its lowest value at 12 hours of drought stress, decreasing by 6.6 times, and then stabilized.

Salt stress treatment led to significant upregulation of four genes (*GmbHLH22, GmbHLH24, GmbHLH98, GmbHLH249*), while the expression levels of other genes did not show significant changes (**Supplementary Figure S4**). The transcription levels of *GmbHLH22* in root, stem, and leaf tissues peaked after 12 hours of salt stress, showing increases of 1.47 times, 1.19 times, and 0.31 times compared to the control group, respectively. *GmbHLH24*’s transcription levels in root and stem tissues also peaked at 12 hours of salt stress, increasing by 9.35 times and 7.73 times, respectively. The transcription levels in leaf tissues reached their peak at 8 hours of salt stress, increasing by 4.59 times compared to the control group. The transcription levels of *GmbHLH98* in root, stem, and leaf tissues peaked at 12 hours of salt stress, with increases of 119.9 times, 310.5 times, and 144 times, respectively, compared to the control group. For *GmbHLH249*, the transcription levels in root and stem tissues peaked at 12 hours of salt stress, increasing by 3.53 times and 1.97 times, respectively. The transcription levels in leaf tissues reached their peak at 8 hours of salt stress, increasing by 0.51 times compared to the control group.

### Subcellular localization

**Figure 7:**
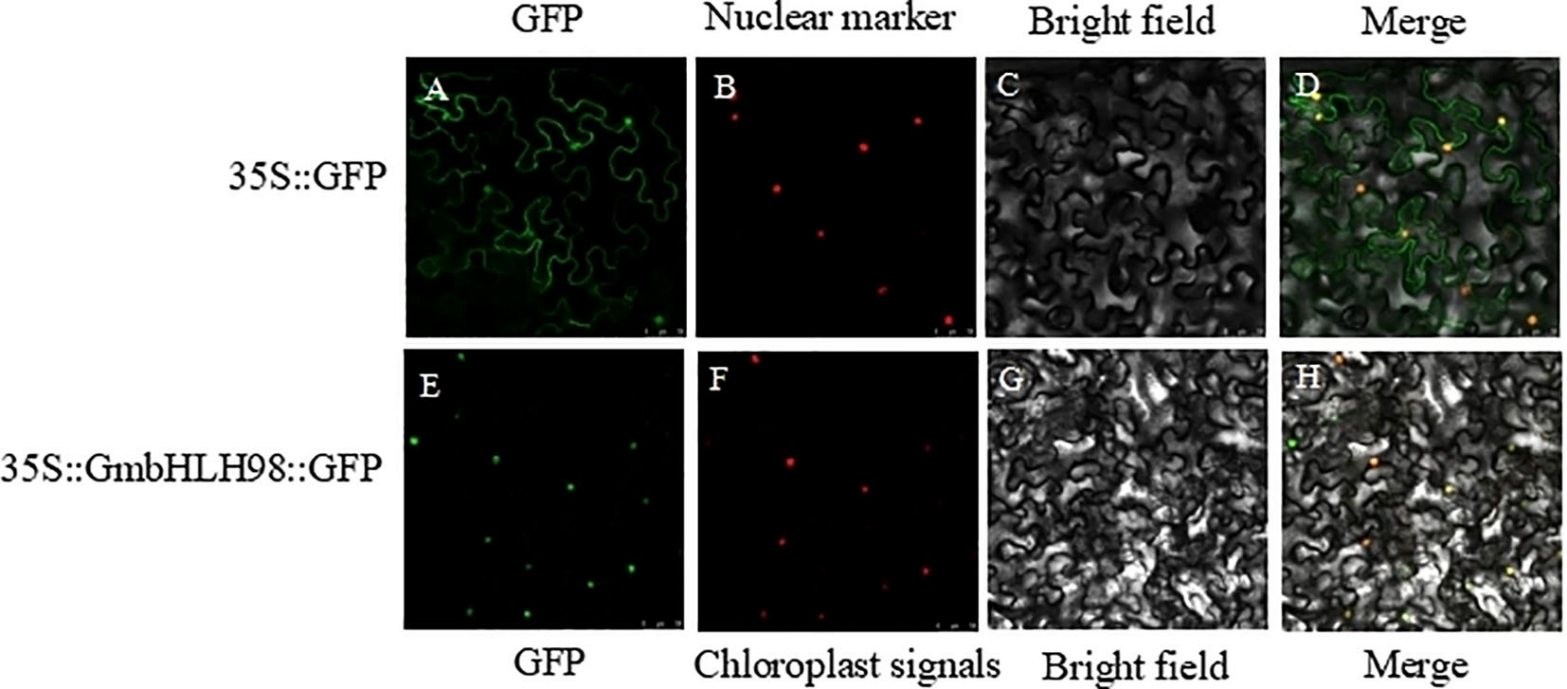
Subcellular localization analysis of *GmbHLH98* proteins in tobacco cells. Scale bar represents 50 mm. **(A, E)** GFP fluorescence; **(B)** Nuclear marker fluorescence; **(F)** chloroplast signals; **(C, G)** Bright-field images; **(D, H)** Merged images. After incubating in the dark for 3 days, the position was observed under a Leica confocal microscope.

The functional analysis of genes largely relies on understanding the localization of gene expression products within cells. To determine the expected subcellular location of *GmbHLH98*, we performed transient expression analysis using pCAMBIA1302-GFP and pCAMBIA1302-GmbHLH98-GFP in tobacco leaves. Green fluorescence signals without GFP connections were observed in the nucleus and cytoplasm of tobacco (**Figure 7**). However, the green fluorescence signal of GmbHLH98-GFP was limited to the nucleus, confirming that *GmbHLH98* is localized in the nucleus of the cell.

### Genetic transformation and fluorescence quantitative PCR detection of T_2_-positive plants

The overexpression vector pCAMBIA3301-GmbHLH98 and the gene editing vector pCRISPR-GmbHLH98, which were successfully constructed earlier, were transferred into the recipient soybean “Jike Soybean 20” using an Agrobacterium-mediated method. PCR was used to detect T2 positive plants, resulting in the screening of a total of 14 positive plants (**Supplementary Figure S5A**). Sequencing results indicated homozygous mutations in the T2 generation of gene-edited plants (**Supplementary Figure S5B**).

Primer design targets *β-ACTIN* as an internal reference gene to detect transcriptional changes in overexpressed and gene-edited materials. Due to a mutation at the target site, the gene exhibits abnormal transcriptional activity, resulting in decreased expression levels. Compared to the control, expression levels of the *GmbHLH98* gene in roots, stems, and leaves have increased to varying degrees. In OE1, expression in roots increased by 3.5 times, in stems by 4.8 times, and in leaves by 5.2 times. In OE2, root expression increased by 5.8 times, stem expression by 7.1 times, and leaf expression by 6.2 times. Conversely, in KO1, root expression decreased by 96.5 times, stem expression by 92 times, and leaf expression by 92.8 times. In KO2, root expression decreased by 22 times, stem expression by 49 times, and leaf expression by 68 times. These findings are illustrated in **Supplementary Figure S6**.

### Dentification of drought and salt tolerance in soybean seedlings transferred to *GmbHLH98* gene

**Figure 8:**
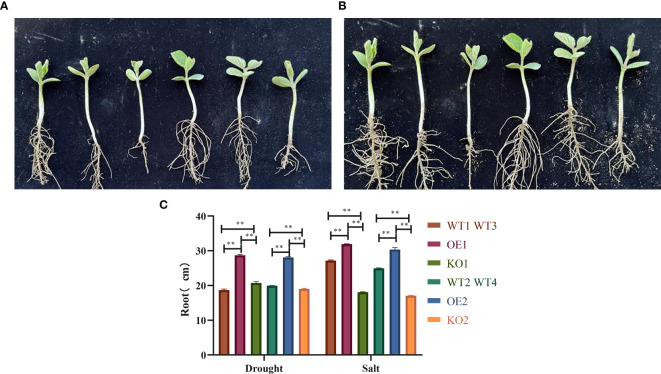
Root development of soybean seedlings with the *GmbHLH98* gene under drought and salt stress: **(A)** Treatment of control varieties with 200 mmol/L PEG6000, overexpression, gene editing, and observation of root development on the 25th day of seedling emergence (V2 stage); **(B)** Treatment of control varieties with 200 mmol/L NaCl, overexpression, gene editing, and observation of root development on the 25th day of seedling emergence (V2 stage); **(C)** Statistical analysis of root length of control varieties, overexpression, and gene editing under 200 mmol/L PEG6000 and 200 mmol/L NaCl, respectively. The data are represented as the average of three values, and the error is represented as SD. ** represents p<0.01.

In an artificial climate chamber simulating drought and salt stress, the root development phenotype of transgenic *GmbHLH98* soybeans was observed during the seedling stage. Under drought stress, root growth in OE1 and OE2 was significantly better than in KO1 and KO2, as shown in **Figure 8A**. After treatment with 200 mmol/L PEG6000, OE1 and OE2 exhibited significantly higher growth potential than the control, while KO1 and KO2 showed significantly lower growth potential. Under salt stress, OE1 and OE2 also demonstrated superior root growth compared to KO1 and KO2, as depicted in **Figure 8B**. Following treatment with 200 mmol/L NaCl, OE1 and OE2 maintained higher growth potential than the control, while KO1 and KO2 showed reduced growth potential, indicating a significant positive regulatory effect of the *GmbHLH98* gene on drought and salt stress resilience (**Figure 8C**).

### Effects of drought stress and salt stress on protein content in soybean seeds

The development of the soybean root system directly impacts its yield and quality. Under drought and salt stress, the stress resistance of different transgenic materials varied. Measurements were taken for the 100-grain weight, plant height, oleic acid, and protein content in overexpression material (OE), control material (WT), and gene-editing material (KO). Results indicated that the overexpression material outperformed the control and gene-editing materials in growth. Trait investigations under normal growth conditions revealed no significant differences in 100-grain weight and plant height among overexpression line (OE), gene-editing plants, and control plants. However, the protein content in OE1 and OE2 increased by 10.42% and 6.12%, respectively. Under drought stress, overexpression line (OE) exhibited significant differences in 100-grain weight and plant height compared to the control (WT) and gene-edited material (KO). The protein content of OE1 and OE2 increased by 10.58% and 17.09%, respectively. Under salt stress, overexpression line (OE) displayed significant differences in grain weight and plant height relative to the control (WT) and gene-edited material (KO). The protein content of OE1 and OE2 increased by 13.83% and 12.78%, respectively, demonstrating that overexpression of *GmbHLH98* can enhance protein content in soybean seeds under stress (**Supplementary Table S5**). A survey on the agronomic traits of the positive material and the control variety Jike Soybean 20 revealed purple flowers, pointed leaves, and gray hairs in both, with no significant differences from the control variety.

**Figure 9:**
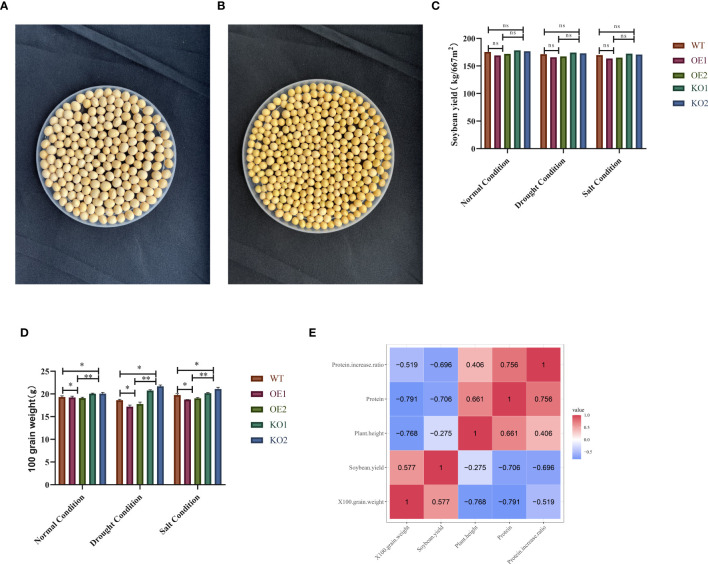
Comparison and overexpression of the phenotypic characteristics of grain size in plants. Under normal harvesting conditions, the grain size and seed coat color of the receptor variety Jike Soybean 20 (WT) were collected at the R8 stage; **(A)** Wildtype (WT) of grain size and seed coat color; **(B)** Overexpression (OE) of grain size and seed coat color; **(C)** Significance analysis of protein content and yield; **(D)** Significance analysis of hundred grain weight and protein content. Data were expressed as the mean of triplicate values and error represented the SD. ** represents p < 0.01. * represents p < 0.05. Non-significant (ns). **(E)** Correlation analysis results between various traits. Protein content was significantly negatively correlated with 100-seed weight (-0.791), plant height was significantly negatively correlated with 100-seed weight (-0.768), yield was significantly positively correlated with 100-seed weight (0.577), protein content was significantly negatively correlated with yield (-0.706), plant height was significantly negatively correlated with yield (-0.275), and protein content was significantly positively correlated with plant height (0.661).

To identify the phenotype of mature seeds from positive materials, we selected overexpressed and receptor seeds for phenotype analysis. Compared to the control variety, the overexpressed offspring exhibited higher protein content, significantly smaller seed size, and darker seed coat color (**Figures 9A, B**). A significant difference analysis revealed no notable difference in yield and protein content between the two groups (**Figure 9C**). However, analysis of hundred grain weight indicated that the overexpressed seeds had higher protein content, smaller grain size, and reduced hundred grain weight (**Figure 9D**). Correlation analysis showed a negative correlation between protein content and yield, with a correlation coefficient of -0.706 (**Figure 9E**).

## Discussion

Most genes in the soybean *bHLH* family are involved in gene duplication events, with gene duplication serving as the primary form of amplification in the *GmbHLH* gene family. By constructing a phylogenetic tree of soybean and Arabidopsis *bHLH* genes, *bHLHs* with similar structures clustered into the same groups. Functionally characterized *bHLHs* in Arabidopsis can provide insights into the roles of closely related *GmbHLHs*. For example, Arabidopsis thaliana *AtNIG1, AtbHLH92*, and *AtbHLH112* regulate salt tolerance. *GmbHLH124, GmbHLH11*, and *GmbHLH8*, which cluster closely with *AtNIG1* (AT5G46830.1), *AtbHLH92* (AT5G43650.1), and *AtbHLH112* (AT1G61660.1), may also regulate salt tolerance (Zhou et al., 2023; Zhang et al., 2022). Overexpression of the soybean *GmbHLH130* gene resulted in significantly longer root lengths under drought treatment compared to the wild type. By contrast, the relative stomatal aperture of transgenic leaves overexpressing *GmbHLH120* was insensitive to ABA, suggesting that *GmbHLH120* may negatively regulate the ABA-mediated drought stress response (Zhou et al., 2023). This study identified numerous tandem and repetitive genes within the *GmbHLH* gene family, indicating that fragment duplication is the main cause of *GmbHLH* gene amplification, consistent with findings in other species such as Arabidopsis and rice. The collinearity of genes serves as a powerful method for analyzing the evolutionary trajectories of genes. In this experiment, collinear genes were identified in rice, corn, and tobacco, suggesting that these homologous genes may share a common ancestor predating their differentiation. In summary, the *GmbHLH* gene is highly conserved in soybean.

The cis-elements of the promoter play a crucial role in regulating gene expression, particularly at the transcriptional level, where the interaction between transcription factors and promoter binding sites is key (Wang et al., 2018). bHLH transcription factors are the second largest family of transcription factors in plants. In model plants such as Arabidopsis thaliana, it has been confirmed that bHLH transcription factors play an important regulatory role in plant growth and development and stress resistance. Research has found that soybeans contain a large number of bHLH transcription factor family members with conserved and specific characteristics. The number of soybean genomes is approximately twice that of Arabidopsis thaliana, corresponding to soybeans being an ancient tetraploid crop. Evolutionary analysis with Arabidopsis thaliana proteins revealed that each family contains members of both soybeans and Arabidopsis thaliana, indicating that these subfamily members are highly homologous in soybeans and Arabidopsis thaliana; Through the analysis of the cis-acting elements in the promoters of soybean GmbHLH family genes, it was found that most members of this family contain E-box sites. *GmbHLH98* can enhance transcription levels and improve its ability to resist abiotic stress and nitrogen fixation by binding to it, which is similar to the results of Zhang et al. (2022). Both wheat *TabHLH1* and rice *OsbHLH035* are known to regulate genes in the ABA signaling pathway and enhance salt tolerance in transgenic plants. Additionally, bHLH proteins can modulate plant drought resistance by influencing ABA and JA signaling pathways (Liang et al., 2022; Joo et al., 2021; Khoso et al., 2022). Notably, among the differentially expressed genes selected for stress response, most of the drought stress-responsive *GmbHLH* genes exhibited positive regulation during drought stress, while a minority showed negative regulation. This highlights the significance of these *GmbHLH* genes in regulating soybean drought tolerance. In cantaloupe, transgenic plants overexpressing *CmbHLH32* demonstrated enhanced lateral root development, root elongation, and increased seed germination potential and rates under stress conditions (Tan et al., 2021). During abiotic stress responses, the expression levels of bHLH transcription factor-related genes significantly increase.

The formation of root nodules is a prerequisite for the establishment of the symbiotic nitrogen fixation system in legumes. Currently, there have been reports on the role of transcription factor bHLH family genes in regulating the process of nodule formation and nitrogen fixation in legumes. Wu analyzed the evolutionary process of the bHLH transcription factor family in legumes and found that there were significant differences compared with the evolutionary process of other plant bHLH families, suggesting that it may be related to the unique nodulation and nitrogen fixation function of legumes (Wu, 2020). Godillard analyzed the transcriptome data during the symbiotic interaction between alfalfa and rhizobia and found that there were differences in the expression of bHLH family transcription factor encoding genes. Further qRT-PCR analysis of gene expression showed that the gene was induced by rhizobia and may be related to nodulation and nitrogen fixation (Godilard et al., 2007). Our research group found in previous research work that under drought and salt stress conditions, the number of soybean roots decreases, root development is poor, and the fresh and dry weight of roots decreases. Zhuang also found that low phosphorus stress significantly reduced the number, fresh weight, nitrogen content, and phosphorus content of soybean nodules (Zhuang et al., 2021). It can be seen that discovering new genes that can improve the ability of soybeans to form nodules and fix nitrogen under abiotic stress conditions is crucial to solving the above problems.

Transcription factors are a class of protein molecules with special structures that participate in gene expression regulation (Francois et al., 2020; Liang et al., 2022; Shen et al., 2021). Like other transcription factors, as a transcription factor involved in resistance to abiotic stress and soybean nodulation, it is necessary to further clarify the mechanism of action involved in the entire regulatory process in the *GmbHLH98*-centered transcriptional regulatory network, as well as the functions of the regulated functional genes or proteins that interact with it, through experiments. This study has preliminarily clarified the function of *GmbHLH98* in soybean resistance to abiotic stress. In the regulatory mechanism of bHLH transcription factors, it is also a question worthy of further attention to predict the role of DNA binding domains in regulating downstream functional genes or interacting with other proteins in *GmbHLH98*.

However, unlike alfalfa, soybean root nodules are stereotyped. During the early symbiotic differentiation of soybeans and rhizobia, Whether it can specifically bind to rhizobia and participate in the formation and development of root nodules like *MtRSD*, and whether there are genes in soybeans that can be regulated by this transcription factor. In the process of soybean-rhizobia interaction, whether *GmbHLH98* can specifically recognize the macromolecular substances produced by rhizobia through interacting with other receptors or protein kinases is also worth further exploration. Due to the specific background of sample selection and data collection, the generalizability of research results may be limited. In addition, the time and data sources of the study may also limit the timeliness and comprehensiveness of the results. In order to further deepen the research, our future studies can expand the sample size to cover more diverse backgrounds and contexts, in order to improve the representativeness of the conclusions.

At present, the functional mechanism of *GmbHLH* gene and its related biosynthesis pathway have been studied, but the specific mechanism is not fully understood. In order to further investigate the molecular mechanism of *GmbHLH98* involved in nodulation and root nodule development, based on the clear physiological and biochemical functions, RNA-seq and ChIP-seq can be used to screen the target genes of *GmbHLH98*, and the target genes screened by omics methods can be further verified by yeast one-hybrid or EMSA; At the same time, through IP-MS combined with high-throughput screening of interacting proteins, the interaction proteins screened were verified by yeast two-hybrid, and the molecular regulatory network at the early stage of nodulation was improved, providing important clues for further exploring the molecular mechanism of soybean *GmbHLH* gene in the synergistic regulation of soybean resistance to abiotic stress and protein synthesis, and also providing a reference for breeding high-yield and high-protein soybean varieties.

## Conclusions

This study identified and cloned members of the soybean *GmbHLH* gene family, including the *GmbHLH98* gene associated with stress response, for the first time. We constructed overexpression and gene editing vectors for the *GmbHLH98* gene, resulting in T2 generation transgenic materials that underwent molecular biology, yield, and quality testing. Overexpression plants significantly improved in the growth and development of main and lateral roots under drought and salt stress compared to control varieties. This suggests that overexpressing the *GmbHLH98* gene can enhance root growth and metabolism, thereby improving plant resistance to stress. Measurements of 100-seed weight, plant height, and protein content were conducted on overexpressing plants, control varieties, and gene-edited plants. Under normal conditions, overexpressing plants showed no significant difference in height but exhibited an increase in protein content. Under drought and salt stress, significant differences were observed in 100-seed weight, plant height, and protein content of overexpressing plants, indicating that these stress conditions benefit soybean protein synthesis, consistent with the findings of Dong (Dong et al., 2021). The variations in hundred grain weight and plant height suggest that bHLH transcription factors, which contain multiple cis-acting elements related to growth, photosynthesis, and substance transport, can influence changes in plant phenotype. Comparison of soybean seed sizes revealed a negative correlation between seed size and protein content. Small soybean seeds yielded less than the control variety but had higher protein content, showing a negative but statistically insignificant correlation. This correlation aligns with the research findings of Fei (Fei et al., 2019) and supports the hypothesis that seed size and protein content, being quantitative genetic traits, have a complex relationship. The experiment provides a theoretical basis and materials for the future cultivation of high-yield and high-protein soybean germplasm. Further research is necessary to explore the underlying mechanisms, particularly considering the differential nutrient transfer and photosynthetic product transportation capacities observed.

## Data Availability

The sequence information for the entire soybean genome was obtained from the Phytozome 13 website (https://phytozome-next.jgi.doe.gov/) and China National Center for Bioinformation (https://ngdc.cncb.ac.cn/soyomics/transcriptome/tissues). Jike Soybean 20 is selected and provided by Jilin Agricultural Science and Technology College. Genome-wide transcriptome data of different soybean tissues were acquired from the NCBI short read archive database as accession LOC100778376.The dataset paper supporting this conclusion is included in this article and its **Supplementary Files**.

## Supplementary material

Supplementary Table 1 Physical and chemical properties of soybean *GmbHLH*.

Supplementary Table 2 KaKs ratio.

Supplementary Table 3 Analysis of the cis acting element of the promoter.

Supplementary Table 4 PCR primers were detected.

Supplementary Table 5 Investigation on the characteristics of T2 generation strains.

Supplementary Figure 1 Collinearity analysis of soybean bHLHs protein family.

Supplementary Figure 2 Analysis of GmbHLHs promoter components.

Supplementary Figure 3 Analysis of expression patterns of GmbHLH genes under drought stress.

Supplementary Figure 4 Analysis of expression patterns of GmbHLH genes under salt stress.

Supplementary Figure 5 PCR detection of positive plants in T_2_ generation. **(A)** PCR detection of positive plants in T_2_ generation. M, DNA DL2000 marker; P, positive control; CK, untransformed plants; 1–8, transformed plants. **(B)** Peak plot of gene edited plant sequencing. **(A, B)** Peak sequencing map of “Jike Soybean 20”; **(C)** Peak sequencing plot of target 1.D.Peak sequencing plot of target 2.Note: The red arrow indicates the editing position, and the editing type is base deletion.

Supplementary Figure 6 Fluorescence quantitative PCR detection of T2 positive plants CK Jike Soybean 20; OE1 and OE2 overexpression materials **(A)**; KO1 and KO2 gene editing materials **(B)**.

Supplementary Figure 7 Transcriptome analysis under different stresses.
