# Supplementary material for: Genome-wide identification and expression analysis of soybean bHLH transcription factor and its molecular mechanism on grain protein synthesis
Source: Front Plant Sci. 2025 Feb 19;16:1481565. doi: 10.3389/fpls.2025.1481565 (PMC11879992; doi:10.3389/fpls.2025.1481565)
Supplement: Supplementary Table 4 — PCR primers were detected. [file Table4.doc]

TABLE S4 Primers Information

| Name | Sequences |
| --- | --- |
| bHLH22-F | CAGCAACTTCGTAACTGGAAC |
| bHLH22-R | CAACTTGAACTTCCACTTTGCT |
| bHLH24-F | CGCAAAGCCTAACCTTATCCAG |
| bHLH24-R | TCGCCGTGGAAGTTCAATTCAA |
| bHLH98-F | TCACAATCCAGGGACCTTAGCT |
| bHLH98-R | AATCGTGGAACTTGCTACGAGT |
| bHLH115-F | GACGTTCGAACTTGCGTGCAGC |
| bHLH115-R | TTCGCATTGCTAGGACCTAACA |
| bHLH188-F | AGGTCCGCTAGGTCAGTTGGA |
| bHLH188-R | GTTGGGCCAAAGTGGAAGTTCT |
| bHLH225-F | ACTTGAACTTCCACTTTGGCCCG |
| bHLH225-R | ATGCGTCGTACCGTAAGTCA |
| bHLH249-F | TCACAACTTACCCTACTGTAGG |
| bHLH249-R | TTGCTACTTCCCTTATAGCCT |
| bHLH250-F | TTAGCTAACGTTGCAATGGC |
| bHLH250-R | ACGGTGCCAGTGGCACTGAA |
| Qβ-ACTIN-F | TGCCTACGTCGTCTAGAACTG |
| Qβ-ACTIN-R | GTGAACGTAGAGGAATGCCA |
| bHLH98:GFP:F | ATCGTTGCGAACGCCTAGA |
| bHLH98:GFP:R | CTTATCTGGATTGGGTTGAC |
| Cas9-98F | CCAGGGATTGCAAG |
| Cas9-98R | CGTGGAAGGCCTAC |
| Bar-1F | AAGTCGGAATCTTAGC |
| Bar-1R | CACGTCCAATGCAATG |
| gRNA-98F1 | ACCTTAGCTAACGTCCAGT |
| gRNA-98R1 | TGCTACGAGTCCTGCAGGT |
| gRNA-98F2 | GTCGTCTACCGAATCAGTA |
| gRNA-98R2 | CACTGACGTTACCGTAAGT |
