# Supplementary material for: Genome-wide identification and expression analysis of soybean bHLH transcription factor and its molecular mechanism on grain protein synthesis
Source: Front Plant Sci. 2025 Feb 19;16:1481565. doi: 10.3389/fpls.2025.1481565 (PMC11879992; doi:10.3389/fpls.2025.1481565)
Supplement: Supplementary Table 5 — Investigation on the characteristics of T2 generation strains. [file Table5.doc]

Table S5 Investigation on the characteristics of T_2_ generation strains

|  | Lines | 100 grain weight(g) | Plant height(cm) | Protein(%) | Protein increase ratio |
| --- | --- | --- | --- | --- | --- |
| Normal Condition | WT | 19.33±0.25b | 98.75±4.71a | 41.53±1.08b | - |
|  | OE1 | 19.21±0.31c | 97.21±3.62a | 45.86±1.02a | 10.42% |
|  | OE2 | 19.07±0.21c | 95.07±4.18a | 44.05±0.98a | 6.12% |
|  | KO1 | 20.05±0.12a | 93.19±3.22a | 40.95±1.01c | -1.48% |
|  | KO2 | 20.08±0.27a | 95.66±2.97a | 38.33±0.75c | -7.84% |
| Drought Condition | WT | 18.62±0.19b | 96.79±3.86b | 40.85±1.17b | - |
|  | OE1 | 17.19±0.37c | 98.21±4.25a | 45.17±1.04a | 10.58% |
|  | OE2 | 17.79±0.41c | 98.83±3.85a | 47.83±0.91a | 17.09% |
|  | KO1 | 20.73±0.20a | 90.65±3.28c | 39.87±0.88c | -2.42% |
|  | KO2 | 21.68±0.29a | 93.29±4.01c | 38.82±0.71c | -4.97% |
| Salt Condition | WT | 19.75±0.31b | 92.89±3.92b | 40.21±1.92b | - |
|  | OE1 | 18.75±0.02c | 94.79±4.01a | 45.77±2.12a | 13.83% |
|  | OE2 | 19.03±0.18c | 95.21±3.75a | 45.35±3.02a | 12.78% |
|  | KO1 | 20.18±0.12a | 90.88±2.01c | 38.12±1.12c | -5.20% |
|  | KO2 | 21.09±0.39a | 91.28±2.92c | 38.79±1.08c | -3.53% |
