# Supplementary figures and images for: Genome-wide identification and expression analysis of soybean bHLH transcription factor and its molecular mechanism on grain protein synthesis
Source: Front Plant Sci. 2025 Feb 19;16:1481565. doi: 10.3389/fpls.2025.1481565 (PMC11879992; doi:10.3389/fpls.2025.1481565)

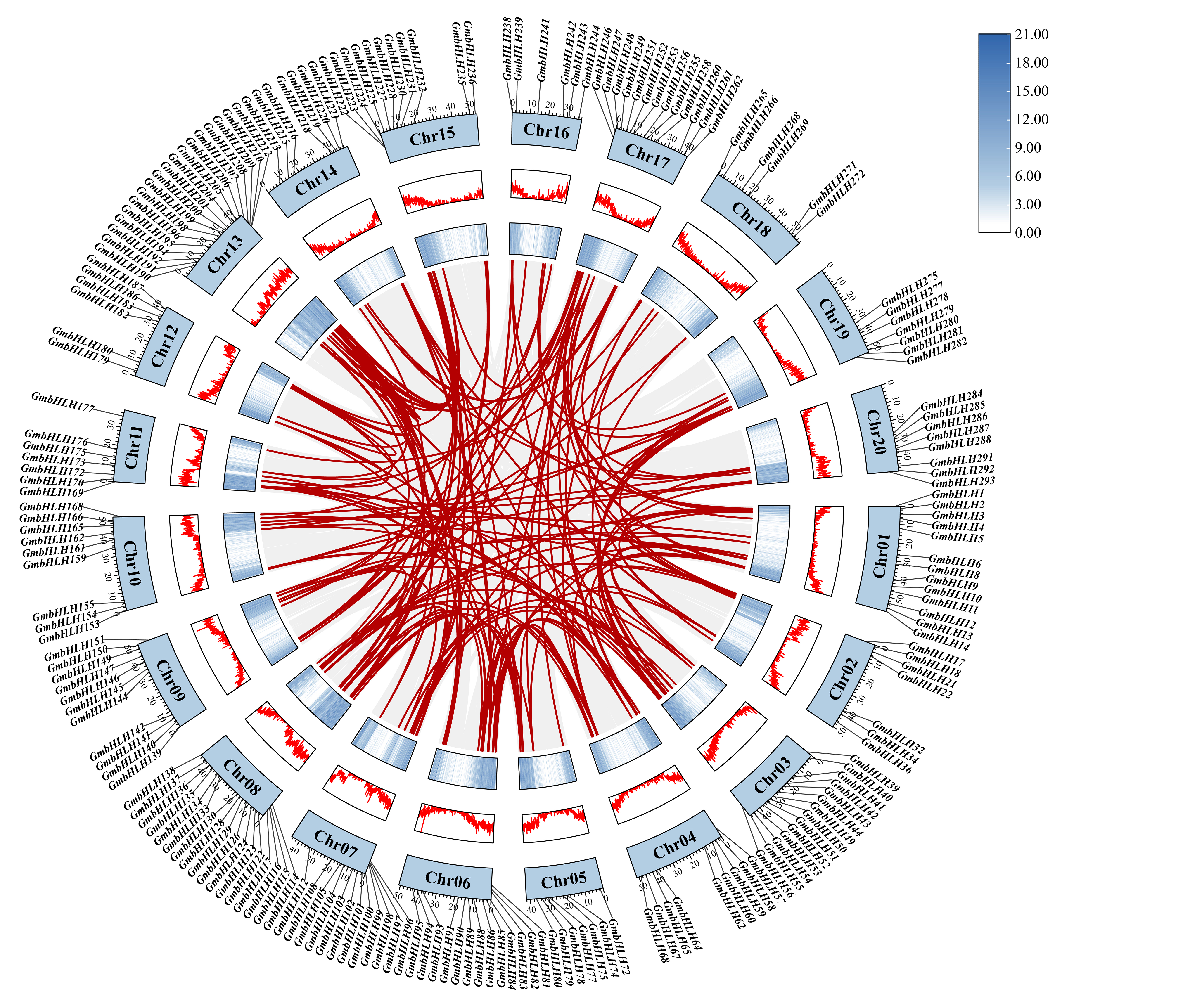

Supplement: Supplementary Figure 1 — Collinearity analysis of soybean bHLHs protein family. [file Image1.jpeg]

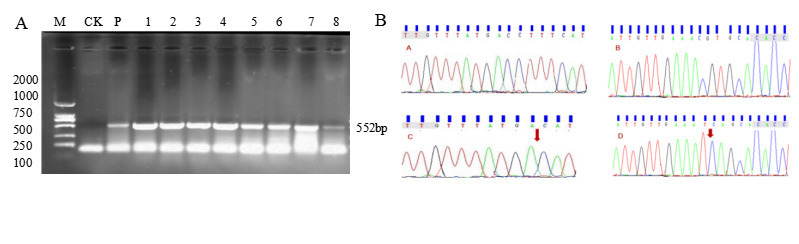

Supplement: Supplementary Figure 3 — Analysis of expression patterns of GmbHLH genes under drought stress. [file Image3.jpeg]

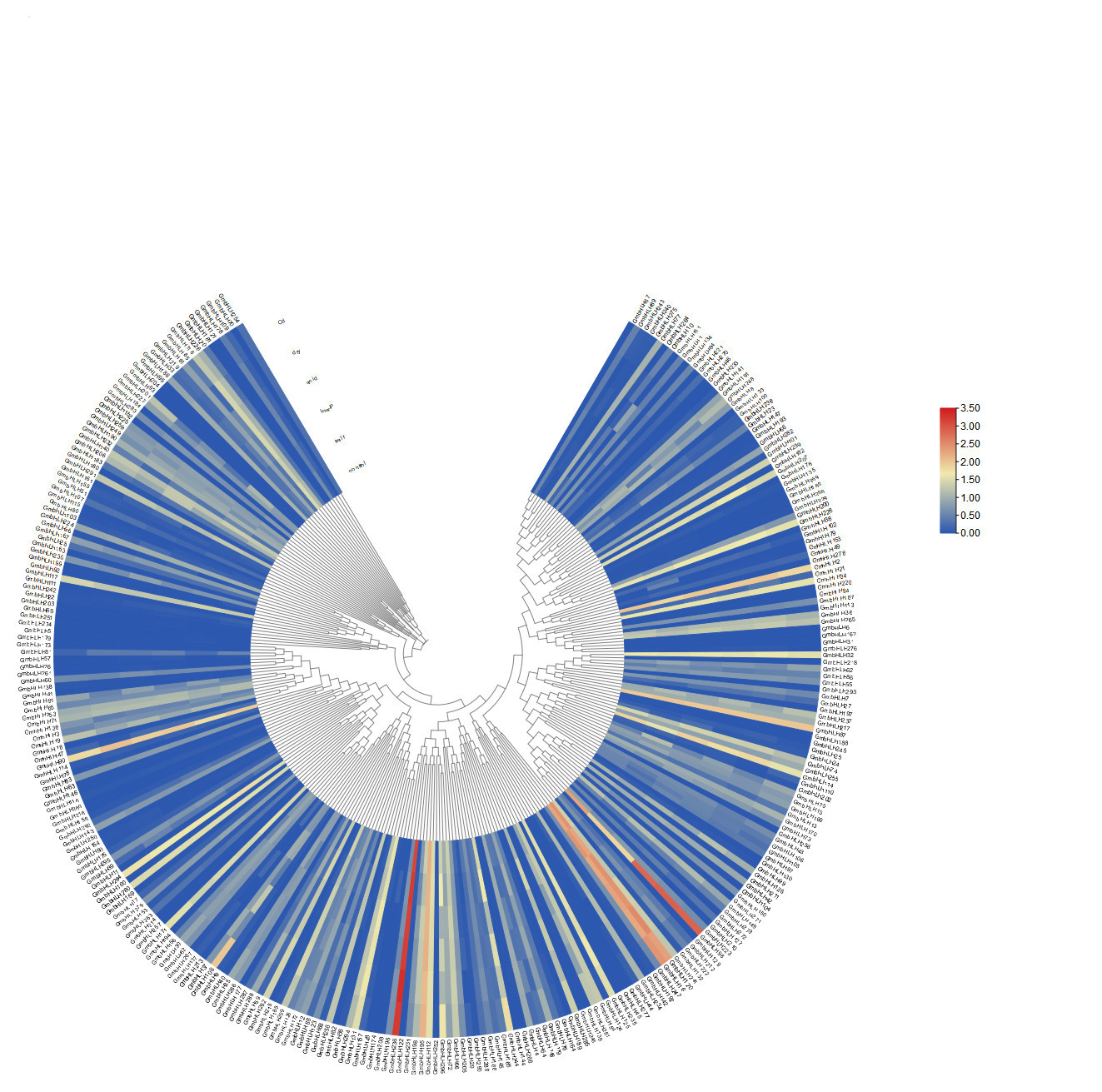

Supplement: Supplementary Figure 4 — Analysis of expression patterns of GmbHLH genes under salt stress. [file Image4.jpeg]

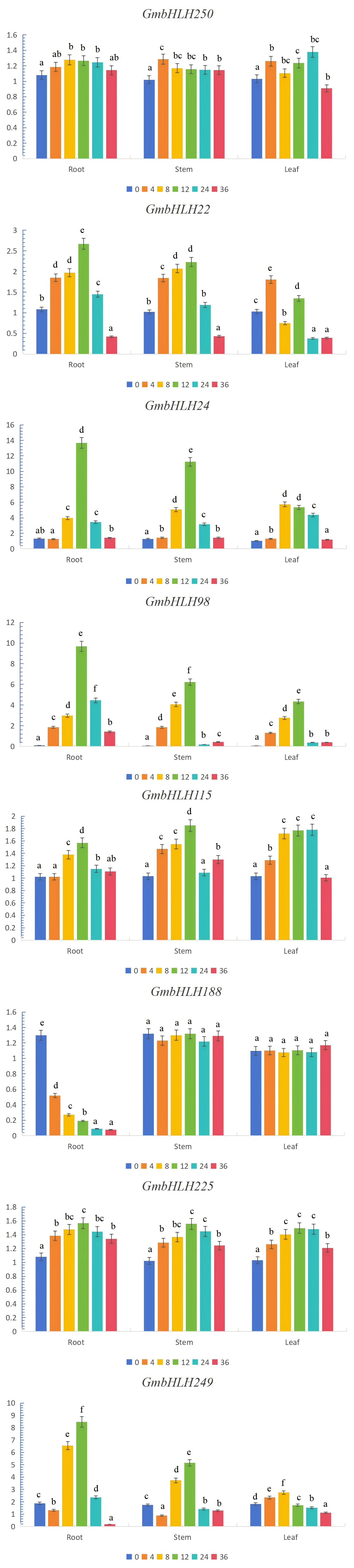

Supplement: Supplementary Figure 5 — PCR detection of positive plants in T2 generation. (A) PCR detection of positive plants in T2 generation. M, DNA DL2000 marker; P, positive control; CK, untransformed plants; 1–8, transformed plants. (B) Peak plot of gene edited plant sequencing. (A, B) Peak sequencing map of “Jike Soybean 20”; (C) Peak sequencing plot of target 1.D.Peak sequencing plot of target 2.Note: The red arrow indicates the editing position, and the editing type is base deletion. [file Image5.png]

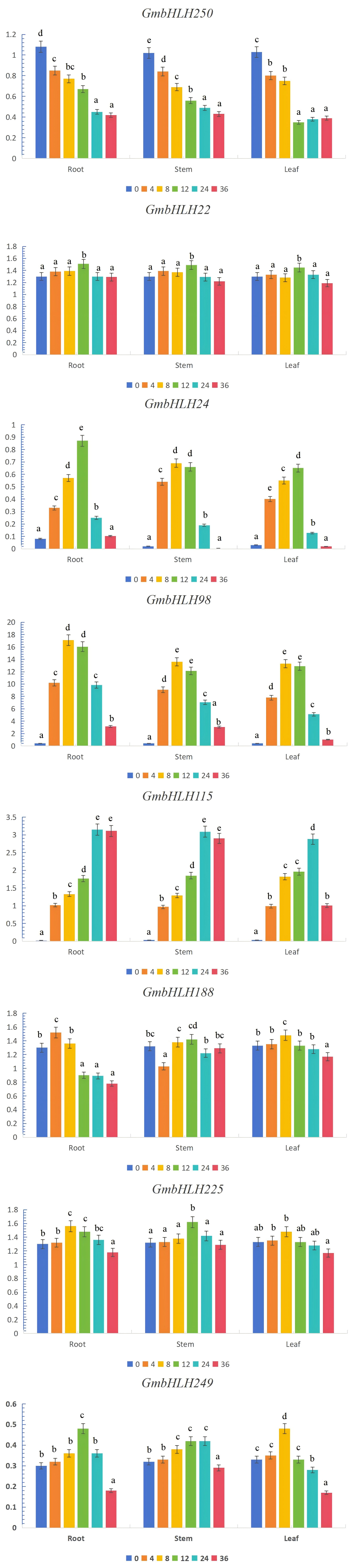

Supplement: Supplementary Figure 6 — Fluorescence quantitative PCR detection of T2 positive plants CK Jike Soybean 20; OE1 and OE2 overexpression materials (A); KO1 and KO2 gene editing materials (B). [file Image6.png]

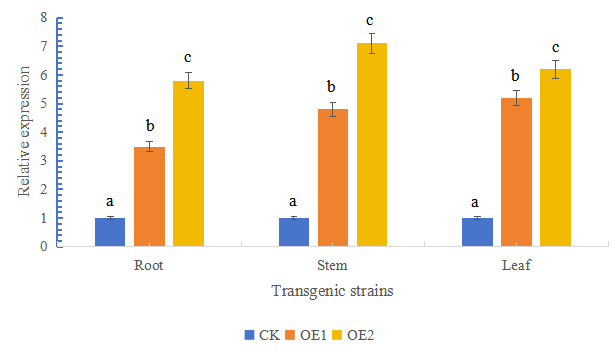

Supplement: Supplementary Figure 7 — Transcriptome analysis under different stresses. [file Image7.png]

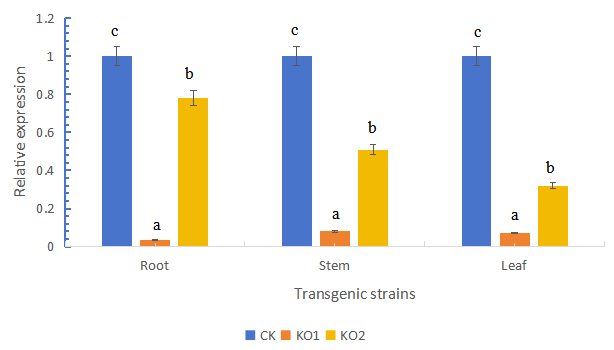

Supplement: Supplementary file 13 [file Image8.png]
